# Learned fear to social out-group members are determined by ethnicity and prior exposure

**DOI:** 10.3389/fpsyg.2015.00123

**Published:** 2015-02-16

**Authors:** Armita Golkar, Marie Björnstjerna, Andreas Olsson

**Affiliations:** Department of Clinical Neuroscience, Psychology Section, Karolinska Institutet, Stockholm, Sweden

**Keywords:** fear learning, extinction, race, ethnicity, preparedness

## Abstract

Humans, like other animals, have a tendency to preferentially learn and retain some associations more readily than others. In humans, preferential learning was originally demonstrated for certain evolutionary prepared stimuli, such as snakes and angry faces and later extended to human social out-groups based on race (Olsson et al., 2005). To address the generality of this social learning bias, we examined if this learning bias extended to two separate classes of social out-groups represented by neutral Black and Middle-Eastern faces in 38 White (Swedish) participants. We found that other-ethnicity alone was not sufficient to induce an out-group learning bias; it was observed for Black, but not Middle-Eastern, out-group faces. Moreover, an exploratory analysis showed that growing up in an ethnically diverse environment was inversely related to the learning bias toward Middle-Eastern, but not Black, out-groups faces, suggesting that learned fears toward Middle-Eastern faces might be more permeable to environmental factors. Future research should address how both the quantity and quality of inter-group contact modulate out-group learning.

## INTRODUCTION

Many intergroup conflicts involve groups defined along social dimensions, such as race/ethnicity, nationality, and religion. A key to understanding the formation of such intergroup relations comes from studying the tendency to learn to favor members of one’s own group (in-group bias) and disfavor those outside of one’s group (out-group bias). In the aftermath of 9/11, the threat of terrorist attacks has become salient to people in Western countries, giving rise to a polarization between the western societies and the Middle East. Previous research has shown that peoples’ negative attitudes are stronger toward Arabs than toward other ethnic groups in both Europe (Snellman and Ekehammar, 2005) and the US (Nosek et al., 2007). In the present study, building upon research demonstrating a bias in learning to fear members of a racial out-group among White and Black Americans (Olsson et al., 2005), we used a classical learning model to compare the formation and persistence of aversive learning toward out-group members of Middle-Eastern origin to that of Black members of African origin in White Europeans.

In classical learning theory, the assumption that all associations are formed and maintained with equal ease was challenged in the 1970s by the evolutionary preparedness theory proposed by Seligman (1971), who emphasized the functional significance of maintaining learned fears to a certain class of so called prepared stimuli in order to avoid imminent threats. This notion has subsequently received empirical support from research in both humans (Öhman et al., 1976) and non-human primates (Mineka et al., 1984). In humans, prepared fear learning has been consistently demonstrated as a persistence in conditioned fear responses to fear-relevant stimuli, such as snakes and angry faces, in comparison to fear-irrelevant stimuli, such as flowers and happy faces (Öhman and Mineka, 2001). More recently, prepared learning was extended to a social category of stimuli by Olsson et al. (2005), who showed a persistence in learned fear to pictures of members of another, as compared with one’s own racial group, among both White and Black Americans. A similar learning bias was subsequently shown in a White Australian population using Asian faces as out-group faces (Olsson et al., 2005) and has been demonstrated to be restricted to male faces (Navarrete et al., 2009). Collectively, these findings point toward the possibility that on a conceptual level, similar learning mechanisms might play a role in the development of fears toward fear-relevant animals and socially relevant stimuli, such as out-group faces. Noteworthy however, fear conditioned to social stimuli, such as angry facial expressions and out-group faces, has been shown to be more permeable to cognitive factors (Olsson et al., 2005; Rowles et al., 2012) than fears conditioned to non-social but fear-relevant stimuli (i.e., snakes; see Mallan et al., 2013 for a recent review on this topic), and has recently been suggested to reflect a more general in-out group differentiation than race alone (Navarrete et al., 2012). These findings suggest that these learning biases might in some cases be malleable. Interestingly however, although a sizable literature suggest that exposure to out-group members should diminish racial biases and negative attitude toward racial out-group members (“contact hypothesis,” Allport, 1954; Pettigrew and Tropp, 2006), and that the degree of out-group pre-exposure may account for the differences in out-group fear learning (“latent inhibition”; Maia, 2009), neither negative racial attitudes or out-group pre-exposure moderated the out-group learning bias toward Black faces among White participants (Olsson et al., 2005; Navarrete et al., 2009).

A pressing question is whether the demonstrated out-group effect is comparable across minority groups in the society. Here, we investigated the persistence of fears acquired to two separate out-group faces by using pictures of Black faces, studied previously in an American context (Olsson et al., 2005; Navarrete et al., 2009) and Middle-Eastern male faces, representing a specifically stigmatized group in an European context (Snellman and Ekehammar, 2005). If representations of in-group and out-group ethnicity are sufficient to induce a learning bias, then fear acquired to faces of both out-groups, but not the in-group, would persist after repeated non-reinforced exposure (i.e., show resistance to extinction). Alternatively, if the previously observed out-group bias toward Black faces as compared to White faces, is not present toward Middle-Eastern faces, this would suggest that this out-group bias may be mediated by factors other than ethnicity alone. To address if out-group learning toward Black faces, as compared to White in-group faces, differs from the out-group learning toward Middle-Eastern faces, as compared to White in-group faces, we used an established within-subjects design for the out-group vs. in-group factor (Olsson et al., 2005; but see Mallan et al., 2009 for a between-subjects design) and introduced out-group ethnicity (Black or Middle-Eastern) as a between-subjects factor. In contrast to previous studies on learning biases toward Black faces that have indexed fear learning using the skin conductance response (SCR; Olsson et al., 2005; Navarrete et al., 2009), we used the fear potentiated startle (FPS) response as our index of fear learning. This was done to address whether the previously observed out-group learning bias toward Black faces was also present using this more basic, brain stem mediated, defensive reflex (Lang et al., 2000). FPS has been successfully used as an index of out-group learning bias in similar research (Mallan et al., 2009).

## MATERIALS AND METHODS

### PARTICIPANTS

Forty-eight participants were recruited through poster advertising on Karolinska Institutet campus. The study was approved by the local ethics committee at Karolinska Institutet, and all participants gave written consent prior to the experiment and were given with two cinema tickets for their participation. We excluded 10 participants of non-Caucasian or Middle-Eastern origin as assessed by a post-experimental interview. The final sample consisted of 38 Caucasian, non-Middle Eastern participants (25 female) of which 17 (mean age = 26.9 years, SD = 8.03) had been randomly assigned to the White/Black group and 21 (mean age = 27.3 years, SD = 7.71) to the White/Middle-Eastern group. In the White/Black group, participants were fear conditioned to a pair of White (in-group) and to a pair of Black (out-group) faces; and in the White/Middle-Eastern group, participants were fear conditioned to a pair of White (in-group) and to a pair of Middle-Eastern (out-group) faces.

### STIMULI

Six different pictures depicting neutral male faces served as CSs; two faces were Central African (Olsson and Phelps, 2004), two faces were North European and two faces were Middle-Eastern (Moroccan) decent (Delgado et al., 2006). Faces from the different categories were matched on ratings of aggressiveness, attractiveness, and masculinity based on pilot data collected in an independent sample prior to the experiment. Stimuli were presented in a pseudo randomized order with the criterion that there could be no more than two trials of the same CS in a row throughout the experiment. A white fixation cross was shown on a black background during the inter-trial intervals (ITIs), the duration of which varied between 11 and 15 s (*M* = 13) throughout the experiment. The experiment was run in a sound-attenuated chamber on a desktop PC with a standard 21-inch cathode ray tube (CRT) monitor. Screen resolution was 800 × 600 pixels and the refresh rate was set to 60 Hz. The experiment was programmed in Presentation 13.1 (Neurobehavioral Systems^1^). Participants viewed pictures at a distance of about 1 m. The US was a 100 ms monopolar DC-pulse electric stimulation (STM200; Biopac Systems Inc.^2^) applied to the participant’s right wrist.

### EXPERIMENTAL TASK

Before the experiment started, the shock level was adjusted individually to be “unpleasant but not painful.” The experiment began with a habituation stage involving three presentations of each CS. During fear acquisition, one stimulus (conditioned stimulus, CS+) from each stimulus category co-terminated with the mild electric shock (unconditioned stimulus, US). Each CS+ was presented nine times during acquisition and each of these presentations was reinforced by a US. The other stimulus from each category (CS–) was presented nine times without shock. Each CS was presented for 6 s, and the coupling between a specific CS and the US and the order of the CS presentation was counterbalanced between participants. Immediately following this acquisition stage, extinction training was initiated and involved 12 repeated presentations of each CS without the aversive US.

### QUESTIONNAIRES

After the experimental task, participants completed measurements of implicit (IAT; Greenwald et al., 1998) and explicit race bias (Olsson et al., 2005) and answered the question “Was the environment you grew up in ethnically diverse? (yes/no)”. Additionally, participants answered the question: “How Swedish do you perceive this person” for each CS in the experiment on a scale from 1 (not at all) to 5 (very much). This measure of “Swedishness” was obtained to address whether there was a negative relationship between the degree to which faces were perceived as Swedish and the out-group learning bias (i.e., the less Swedish they were perceived as, the more out-group bias expressed). To address this, we used the ratings for the Swedish faces as a reference category (i.e., out-group ratings were within-subjects controlled for the Swedish in-group ratings) and correlated these ratings against the out-group learning bias (within-subjects controlled for in-group learning). Five participants (2 in the White/Black and 3 in the White/Middle-Eastern group) did not complete these questionnaires and are therefore not included in the analyses based on questionnaire data.

### PSYCHOPHYSIOLOGICAL ASSESSMENT

To elicit fear-potentiated startle responses, a 50 ms burst of white noise (95 dB) with a near instantaneous rise time (<1 ms) served as the startle probe and was presented binaurally over headphones (Sony MDR-CD 170). In each trial, the startle probe was presented 4–5 s following CS onset. Startle probes were presented on an equal number of trials of each CS (1 out of 3 during habituation, 6 out of 9 presentations during acquisition, and on 9 out of 12 presentations during extinction) and during an equal number of ITIs (1 during habituation, 6 during acquisition and 9 during extinction), which served as a baseline. The eye-blink component of the startle response was measured through electromyographic (EMG) recordings of the left orbicularis oculi muscle using two miniature Ag/AgCl electrodes prepared with electrolyte gel. A third ground electrode was placed behind the left ear over the mastoid. The raw EMG signal was amplified and filtered through a 28–500 Hz bandpass filter and integrated with a time constant of 20 ms. Startle eye-blink magnitude (microvolts) was measured as the amplitude from onset to peak (20–120 ms post-probe onset corrected for pre-probe baseline) and normalized using T-standardization. Trials with excessive baseline activity or recording artifacts were discarded following previous procedures (e.g., Weike et al., 2007). Mean startle difference scores for each stage of the experiment (acquisition and extinction) were calculated as [mean startle magnitude during the CS]—[mean startle magnitude during ITI for each session] as has been described previously (Norrholm et al., 2008; Golkar and Öhman, 2012).

### STATISTICAL ANALYSIS

Each session of the experiment (acquisition and extinction) was analyzed separately in a 2 (CS+, CS–) × 2 (In-group face, Out-group face) × 2 (White/Black group, White/Middle-Eastern group) mixed analysis of variance (ANOVA). We adopted a significance level of 0.05 and report as the estimate of effect size. Greenhouse-Geisser adjustments of degrees of freedom were used when appropriate. Significant interactions and pre-planned comparisons were followed up with separate two-tailed *t*-tests.

## RESULTS

**FIGURE 1:**
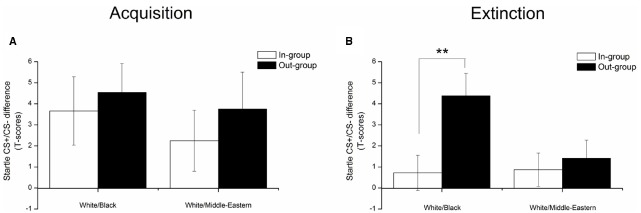
**Acquisition (A) and Extinction (B) of conditioned fear as a function of social group.** The difference between in-group and out-group categories emerged during extinction training and was manifested as a resistance to extinction to Black out-group CSs specifically. For simplicity, the Y-axis represents mean startle CS+/CS– differentiation scores after controlling for baseline (ITI) startle. Error bars represent mean standard error (SEM). **p < 0.01.

The mean differential (CS+ minus CS–) FPS responses during acquisition and extinction are presented in Figure 1. We first confirmed that participants in the White/Black and White/Middle-Eastern group acquired comparable levels of fear to both in-group and out-group faces, as evident by larger FPS responses to the CS+ than the CS– [main effect of CS *F*(1,36) = 13.55, *p* = 0.001; η^2^ = 0.27]. During extinction, fear acquired to Black faces extinguished slower than to Middle-Eastern faces [CS × In/Out group face × Group: *F*(1,36) = 5.10, *p* = 0.03; η^2^ = 0.12]. Thus, whereas fear conditioned to Black out-group faces resisted extinction relative to White in-group faces [CS × In/Out group face, *F*(1,16) = 12.10, *p* = 0.03; η^2^ = 0.43], there were no significant difference in extinction of conditioned fear to Middle-Eastern out-group faces relative to White in-group faces [CS × In/Out group face, *F*(1,20) = 1.5, *p* = 0.56]. Similar to a previous finding (Navarrete et al., 2009) the inclusion of participant sex as a covariate in these analyses did not significantly alter our findings, and neither implicit nor explicit racial attitudes were significantly related to the differential FPS response toward in and out-group CSs during either acquisition or extinction.

Additionally, post-experimental data established that rated degree of “Swedishness” differed between stimulus ethnicities across both experimental groups [main effect of ethnical category: *F*(2,64) = 2015.03, *p* < 0.001; η^2^ = 87]; Black faces were rated as less Swedish than both Middle-Eastern [*t*(32) = 2.95, *p* = 0.006] and White faces [*t*(32) = 15.92, *p* < 0.001] and Middle-Eastern faces were rated as less Swedish than the White faces [*t*(32) = 15.08, *p* < 0.001]. Interestingly, whereas the degree of rated “Swedishness” did not correlate with the out-group learning bias in the White/Black group (*r* = 0.05, *p* = 0.85), lower “Swedishness” ratings predicted stronger out-group learning bias in the White/Middle-Eastern group (*r* = –0.49, *p* = 0.04).

### SUPPLEMENTAL RESULTS

**FIGURE 2:**
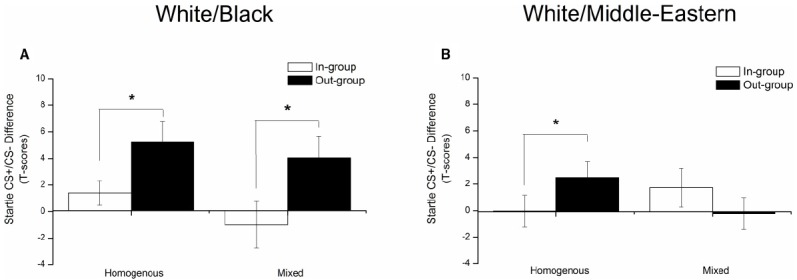
**Extinction of conditioned fear toward in and out-groups as a function of ethnical diversity in upbringing environment displayed separately for (A) White and Black faces and (B) White and Middle-Eastern faces.** Out-group responses toward Black faces survived extinction independently of ethnical diversity in the upbringing environment (*N* = 9 homogenous environment; *N* = 6 mixed environment), whereas out-group responses toward Middle-Eastern faces survived extinction only in participants raised in ethnically homogenous (i.e., European) environments (*N* = 8 homogenous environment; *N* = 8 mixed environment). The Y-axis represents mean startle CS+/CS– differentiation scores after controlling for baseline (ITI) startle. Error bars represent mean standard error (SEM). *p < 0.05.

Finally, to analyze the possible relationship between the learning bias and exposure to a mixed ethnic environment during upbringing, we ran an exploratory analysis comparing conditioned responses (CR; CS+ minus CS–) toward in- and out-group CSs based on whether participants classified their upbringing environment as ethnically mixed or not. Although no significant effects emerged during acquisition learning (all *F*’s < 1.18), extinction of out-group responses differed between the White/Black and White/Middle-Eastern groups as a function of Environment [In/Out group CR × Environment × Group: *F*(1,27) = 5.11, *p* = 0.03; η^2^ = 16]. Thus, as shown in Figure 2, whereas the out-group learning bias toward Black faces was not significantly altered by ethnical diversity in the upbringing environment [main effect of in/out-group CR: *F*(1,13) = 16.01, *p* = 0.001; η^2^ = 55], upbringing environment played a significant role in the group exposed to the Middle-Eastern faces [In/Out-group CR × Environment: *F*(1,14) = 11.75, *p* = 0.004; η^2^ = 0.46]. Follow-up analysis within the White/Middle-Eastern group showed that only participants who rated their upbringing environment as ethnically homogenous, showed the predicted out group learning bias toward Middle-Eastern out-group faces [*t*(7) = 3.29, *p* = 0.01].

## DISCUSSION

Here, we demonstrate a greater resistance to extinction of learned fear toward Black, but not Middle-Eastern, out-group faces. The notion that humans, like other animals, have a tendency to preferentially learn and retain some associations more readily than others has important implications for understanding social behavior; it provides a potential mechanism to understand the emergence and maintenance of social biases. In humans, such preferential learning was originally demonstrated for a specific class of evolutionary prepared stimuli, such as snakes (Öhman et al., 1976) and angry facial expression (Dimberg and Öhman, 1996), and later extended to human social groups based on race (Olsson et al., 2005; Mallan et al., 2009). More recently, a similar learning bias has been shown to groups defined by more transient (“minimal”) characteristics (Navarrete et al., 2012), suggesting that a more general out-group bias might support the social group findings. In the first study to compare out-group learning to different ethnical groups, we observed a greater resistance to extinction of learned fear toward Black, but not Middle-Eastern, out-group faces. Unlike previous demonstrations of a learning bias toward Black faces that have used SCR, we used the FPS reflex as a measure of learning. The FPS, which is a brain-stem mediated defensive response, has been argued to be a more basic measure of fear learning, and potentially less influenced by cognitive factors, as compared to SCR (Hamm and Weike, 2005). Our replication of the resistance of extinction to Black out-group faces using a new measure of learning further validates this effect. Noteworthy, there was a descriptive trend indicating more pronounced acquisition of CR across both classes of out-groups. A similar trend was present in the original study by Olsson et al. (2005) and more recently reported by Navarrete et al. (2009). Indeed, according to classical conditioning theory, prepared associations are indicated by either superior acquisition or the enhanced resistance to extinction (reviewed by Öhman and Mineka, 2001). Although both types of learning biases have been argued to serve adaptive functions, there is no strong rationale to consider either index as inherently more valid than the other (Rescorla, 1980).

Our findings suggest that the previously observed out-group learning bias toward Black faces in White participants (Olsson et al., 2005; Mallan et al., 2009; Navarrete et al., 2009) does not necessarily generalize across different ethnical groups. Rather, the effect may be mediated by factors other than ethnicity alone. The out-group bias to Black faces in White participants has previously been shown to be unrelated to out-group pre-exposure, although other research has identified intergroup contact (Allport, 1954; Pettigrew and Tropp, 2006) and the degree of pre-exposure to out-group faces (Maia, 2009) as critical to explain out-group biases more generally. Therefore, in an exploratory analysis, we assessed the effects of ethnical diversity in the upbringing environment on the out-group learning bias. Consistent with earlier studies (Olsson et al., 2005; Navarrete et al., 2009), there was no relationship between the diversity of the up-bringing environment and the observed learning bias to Black faces. Interestingly, however, we found that ethnical diversity in the upbringing environment moderated extinction of learned fear to Middle-Eastern out-group faces, suggesting that learned fears of Middle-Eastern faces might be more permeable to environmental factors than learned fears of Black out-group faces. Whereas individuals that had been brought up in a homogeneously White European environment displayed a resistance to extinction to Middle-Eastern faces similar to that of the participants exposed to Black out-group faces, individuals growing up in an ethnically mixed environment did not display an extinction bias to Middle-Eastern out-group faces. Although the small sample size in this analysis requires that this effect is interpreted with caution, the specificity of this finding is intriguing in light of the fact that individuals with a Middle-Eastern origin have been shown to represent a particularly stigmatized social group (Snellman and Ekehammar, 2005; Nosek et al., 2007).

The Black minority is by far outnumbered by the Middle-Eastern minority in the Swedish context in which the study was performed (Statistics Sweden, 2010), and this difference in out-group frequency may be relevant for the interpretation of our findings. Thus, given that it is reasonable to assume that participants raised in ethnically mixed environments are more likely to have been raised among Middle-Eastern individuals rather than among Black African individuals, this differences might contribute to the stronger influence of ethnical diversity on the observed learning bias toward Middle-Eastern out-group faces. Such an explanation would be in line with the latent inhibition account previously offered for similar learning biases (Maia, 2009). Accordingly, the superior learning to social out-group faces results from less prior exposure to individuals belonging to these out-groups. Interestingly, although exposure to out-group members (i.e., growing up in an ethnically mixed environment) was unrelated to the learning bias in the original study (Olsson et al., 2005), the experience of intimate contact (e.g., dating) moderated the effect (Olsson et al., 2005; Navarrete et al., 2009)^3^. Given that growing up in an ethnically mixed environment not only increases mere intergroup exposure but also the chances of intimate contact, it remains possible that the quality, rather than the quantity, of intergroup experience explains the influence of environment on the learning bias toward Middle-Eastern faces in the present study. Another possible determinant of the learning bias is the degree of “in-groupness” (operationalized as “Swedishness”), which in our sample was inversely related to the learning bias in the Middle-Eastern group, i.e., the less Swedish the Middle-Eastern faces were perceived, the more out-group learning bias expressed. Perceptual features such as skin color and facial physiognomy are other possible determinants to the difference in resistance to extinction of fear to Black faces. However, previous findings of similar extinction effects to fear conditioned facial images drawn from other ethnic groups, such as White faces among Black participants (Olsson et al., 2005); Chinese faces among White participants (Mallan et al., 2009), and no effect of Black female faces among White male and female participants (Navarrete et al., 2012), suggest that skin color and facial physiognomy alone or together cannot explain the entire effect. Nevertheless, it remains to be addressed whether our findings generalize to Black and Middle-Eastern participants exposed to White out-group faces. Moreover, although our design allows for a direct between-subjects comparison of out-group learning across two different ethnical groups, it remains unknown whether there is a difference in *relative* out-group learning between Black and Middle-Eastern faces. This possibility can only be tested in a full within-subjects design including two sets (corresponding to the CS+, and the CS–) of Black, Middle-Eastern and White faces. Finally, consistent with previous data on out-group learning biases toward male Black faces (Olsson et al., 2005; Navarrete et al., 2009), we did not observe an effect of participant sex.

To conclude, facial representations of other-ethnicity alone is not sufficient to induce an out-group learning bias, which in our sample of White Europeans was observed for Black, but not Middle-Eastern, out-group faces. However, in individuals growing up in a homogeneously White environment, the learning bias toward Middle-Eastern out-groups faces paralleled the bias expressed toward Black out-group faces, suggesting that learned fears toward Middle-Eastern faces might be more permeable to environmental factors. Given the difference in the frequency of these separate out-group faces in the general population in which testing occurred, important future research questions will be to examine how both the quantity and quality of intergroup contact modulate learning about individuals with a different ethnical background than one’s own, and if the effects of such intergroup contact generalize across ethnical out-groups.

### Conflict of Interest Statement

The authors declare that the research was conducted in the absence of any commercial or financial relationships that could be construed as a potential conflict of interest.

## Footnotes

1 www.neurobs.com

2 www.biopac.com

3 Due to the small number of participants with dating experience with Black individuals (*N* = 5), we did not analyze the effect of dating experience in the present sample.
